# Lineage-specific evolution of the vertebrate *Otopetrin *gene family revealed by comparative genomic analyses

**DOI:** 10.1186/1471-2148-11-23

**Published:** 2011-01-24

**Authors:** Belen Hurle, Tomas Marques-Bonet, Francesca Antonacci, Inna Hughes, Joseph F Ryan, Evan E Eichler, David M Ornitz, Eric D Green

**Affiliations:** 1 Genome Technology Branch, National Human Genome Research Institute, National Institutes of Health, (50 South Drive), Bethesda, MD (20892), USA; 2 Department of Institut de Biologia Evolutiva (UPF/CSIC), (Dr. Aiguader, 88), Barcelona (08003), Spain; 3 Department of Genome Sciences and Howard Hughes Medical Institute, University of Washington School of Medicine, (3720 15th Ave NE), Seattle, WA (98195), USA; 4 Department of Child Neurology, University of Rochester Medical Center, (601 Elmwood Avenue), Rochester, NY (14642), USA; 5 NIH Intramural Sequencing Center, National Human Genome Research Institute, National Institutes of Health, (5625 Fishers Lane), Bethesda, MD (20852), USA; 6 Department of Molecular Biology and Pharmacology, Washington University School of Medicine, (660 South Euclid Avenue), St. Louis, MO (63110), USA

## Abstract

**Background:**

Mutations in the *Otopetrin 1 *gene (*Otop1*) in mice and fish produce an unusual bilateral vestibular pathology that involves the absence of otoconia without hearing impairment. The encoded protein, Otop1, is the only functionally characterized member of the Otopetrin Domain Protein (ODP) family; the extended sequence and structural preservation of ODP proteins in metazoans suggest a conserved functional role. Here, we use the tools of sequence- and cytogenetic-based comparative genomics to study the *Otop1 *and the *Otop2-Otop3 *genes and to establish their genomic context in 25 vertebrates. We extend our evolutionary study to include the gene mutated in Usher syndrome (USH) subtype 1G (*Ush1g)*, both because of the head-to-tail clustering of *Ush1g *with *Otop2 *and because *Otop1 *and *Ush1g *mutations result in inner ear phenotypes.

**Results:**

We established that *OTOP1 *is the boundary gene of an inversion polymorphism on human chromosome 4p16 that originated in the common human-chimpanzee lineage more than 6 million years ago. Other lineage-specific evolutionary events included a three-fold expansion of the *Otop *genes in *Xenopus tropicalis *and of *Ush1g *in teleostei fish. The tight physical linkage between *Otop2 *and *Ush1g *is conserved in all vertebrates. To further understand the functional organization of the *Ushg1-Otop2 *locus, we deduced a putative map of binding sites for CCCTC-binding factor (CTCF), a mammalian insulator transcription factor, from genome-wide chromatin immunoprecipitation-sequencing (ChIP-seq) data in mouse and human embryonic stem (ES) cells combined with detection of CTCF-binding motifs.

**Conclusions:**

The results presented here clarify the evolutionary history of the vertebrate *Otop *and *Ush1g *families, and establish a framework for studying the possible interaction(s) of *Ush1g *and *Otop *in developmental pathways.

## Background

Although bilateral vestibular pathology is an important cause of imbalance in humans, it is underdiagnosed and poorly understood. Approximately 50% of dizziness is attributed to benign paroxysmal positional vertigo (BBPV), a major risk factor for falls, bone fractures, and accidental death, particularly in the elderly [1]. The etiology of BBPV often relates to the degeneration or displacement of otoconia [2,3], the minute biomineral particles in the utricle and saccule within the inner ear involved in detecting linear acceleration and gravity [4].

Currently, there are no known human hereditary vestibular disorders attributed to mutations in genes selectively affecting otoconial development or maintenance. Otopetrin 1 (*Otop1*) is one of a handful of known genes that, when mutated, cause an imbalance phenotype with selective otoconial involvement in animal models [4,5]. In the mouse, *Otop1 *is expressed in the supporting cells of the sensory epithilium patches (maculae) of the utricule and saccule from embryonic day 13.5 to adulthood, as well as in several other tissues [6]. In contrast, *Otop1 *expression in zebrafish is predominantly restricted to the hair cells of the sensory epithelium and the neuromasts of the lateral line organ [7,8]. Specific *Otop1 *mutations have been reported in mouse (*tilted *[*tlt*, [9]], *mergulhador *[*mlh*, [10]], and *inner ear defect *[*ied*, [11]]) and zebrafish (*backstroke *[*bks*, [8]]). Despite the inter-species differences in *Otop1*-expression patterns, mutant mice and fish lack otoconia and otoliths respectively in all cases, but otherwise have normal inner ear structures, hearing, and overall development. Similarly, the knockdown of *Otop1 *in mouse and zebrafish results in a phenocopy of the selective lack of otoconia/otoliths seen in the mutants [6,7].

The *Otop *gene family in most vertebrates is comprised of three members clustered on two chromosomes: *Otop1 *(e.g., mouse chromosome 5) and the paralogous tandem genes *Otop2 *and *Otop3 *(e.g., mouse chromosome 11). The origins of the *Otop *gene family have been traced far back in metazoans [12]; for example, the phylogenetic relationships of vertebrate Otop and arthropod and nematode Otop-like proteins (also DUF270 proteins [10,12]) have been deduced from 62 open reading frames in 25 species, demonstrating that they constitute a single family, named the Otopetrin Domain Protein (ODP) family. Fragmentary, but clearly ODP-related, sequences have also been identified in urochordates (*Ciona*), echinoderms (urchin), and cnidarians (*Nematostella*) (unpublished data). Signature features of ODP proteins are 12 transmembrane domains organized into three "Otopetrin Domains" (highly conserved among metazoans) and a highly constrained predicted loop structure. Although ODP proteins do not show homology to any transporter, channel, exchanger, or receptor families, the extensive sequence and structural similarity among them suggests a conserved functional role(s) [12]. It has been postulated that Otop1 inhibits P2Y purinergic receptor-mediated calcium release in macular epithelial cells in a calcium-dependent manner, and promotes an influx of calcium in response to ATP during otoconial development [6,13]. In this model, Otop1 acts as a sensor of the extracellular calcium concentration near supporting cells, and responds to ATP in the endolymph to increase intracellular calcium levels during otoconia mineralization. Otop2 and Otop3 functions remain unknown.

The larger syntenic context of the *Otop1 *and tandem *Otop2-Otop3 *genomic loci may reflect lineage-specific genomic features and gene associations worthy of exploration. For instance, established genetic and physical maps of the *Otop1*-containing region suggest an inverted gene order in the mouse and human genomes [14,15]; further, in these genomes, the *Otop2*-*Otop3 *gene tandem is physically clustered (head-to-tail) with the Usher syndrome (USH) subtype 1G gene (*USH1G*; also called *SANS*). *USH1G *encodes a scaffold protein with three ankyrin repeats and a SAM domain, which is preferentially expressed in tissues containing motile cilia, such as cochlea and retina [16,17]. Mouse *Ush1g *on chromosome 11 and its human ortholog *USH1G *on chromosome 17 are mutated in the *Jackson shaker *(*js*) mouse mutant and in human *USH1G *patients, respectively. The former is a recessive condition associated with deafness, circling, and disorganization of the hair cell bundles (stereocilia) [16]; the latter is also a recessive condition characterized by congenital deafness, vestibular dysfunction, and prepubertal onset of visual loss (retinitis pigmentosa) [18]. USH is generally a clinically and genetically heterogeneous disease and the most common cause of hereditary deaf-blindness in humans [19]; invariably, USH is associated with highly disorganized stereocilia.

*Ush1g *and *Otop1 *thus play important roles in the vestibular (*Ush1g *and *Otop1*), auditory (*Ush1g*), and visual (*Ush1g) *systems in vertebrates. In addition to the experimentally confirmed expression of *Ush1g *in the mouse and human inner ear and retina [16,17] and of *Otop1 *in the mouse and zebrafish inner ear [7,10], an *Otop*-like gene (*Nlo*, for neural crest-lateral otic vesicle localization) is expressed in the anterior placodal ectoderm area of the *X. laevis *embryo that forms both the eye lens and otic vesicle neurogenic placodes [20]. Furthermore, analyses of expressed-sequence tag (EST) databases reveal evidence for expression of *Otop1*, *Otop2*, and *Otop3 *in the mouse and human retina. The challenge remains to establish whether *Ush1g *and *Otop *participate in common developmental pathways affecting ear and/or eye physiology.

To examine the evolutionary diversity of *Otop *and *Ush1g *and their larger genomic context in vertebrates, we performed a comprehensive comparative genomic study that analyzed 25 available genome sequences (from fish to human) and sequenced bacterial artificial-chromosome (BAC) clones from seven species. Through cytogenetic and comparative genomic methods, we established that *OTOP1 *is the boundary gene of an inversion polymorphism on human chromosome 4p16 that originated in the common human-chimpanzee lineage more than 6 million years ago. We could further infer evolutionary scenarios for gene family expansions in individual lineages, including a three-fold expansion of the *Otop *family in *Xenopus tropicalis *and the *Ush1g *family in fish lineages. *Ush1g *genes have remained tightly linked to *Otop2*-related sequences throughout vertebrate evolution, raising questions about whether they are functionally insulated or whether they participate in common developmental pathways. To further understand their functional organization, we deduced and analyzed a map of putative CCCTC-binding factor (CTCF)-binding sites within the *Ushg1-Otop2 *locus. These analyses offer some hints about chromatin organization of the *Ush1g-Otop2 *locus in mouse and human, and are a resource for further investigating the role of mammalian insulator CTCF in orchestrating gene expression at this locus.

## Results

### Comparative sequence data sets

We sought to characterize the genomic regions encompassing *Otop1 *and the *Ush1g-Otop2-Otop3 *cluster in a diverse set of vertebrates. As the reference sequence for these studies, we extracted relevant portions of the NCBI mouse genome sequence (build 37; [21]), specifically, the intervals containing *Otop1 *on chromosome 5B2 and *Ush1g-Otop2-Otop3 *on chromosome 11E2, plus the 100-kb segments immediately flanking each interval (see Materials and Methods). The human sequence was an unsuitable reference because the human *OTOP1 *locus diverged significantly from the orthologous regions in most other vertebrate genomes (see below).

We compiled the homologous sequences of these two genomic regions from 25 vertebrate species for comparative analyses (Table 1). For seven species, we isolated [22] and sequenced [23] BAC clones spanning one or both of the targeted genomic regions (Table 2); these efforts generated ~3.8 Mb of high-quality sequence data [24] specifically for this study. For the remaining species (and in cases where we were unable to isolate suitable BACs from the species listed in Table 2), the orthologous sequences were retrieved from whole-genome sequence assemblies available on the UCSC Genome Browser (http://genome.ucsc.edu). With few exceptions (i.e., hedgehog and platypus), the generated data sets contain orthologous sequences of the *Otop1 *and the *Ush1g-Otop2-Otop3 *loci from multiple phylogenetic lineages.

**Table 1:** Coordinates for the *Otop1 *and *Ush1g-Otop2-Otop3 *loci on the UCSC Genome Browser (genome.ucsc.edu).

Group	Species	Name	Assembly	Build	*Otop1 *locus	*Ush1g-Otop2- Otop3 *locus
Placental mammals						
Primate	Human^a^	*Homo sapiens*	hg17	v.35.1	Chr4:4309000-4438200^a^	Chr17:70368500-70574600
					Chr4:9457200-9718300^a^	
Primate	Chimpanzee^a^	*Pan troglodytes*	panTro2	v.2.1	Chr4:4274000-4409000^a^	Chr17:74415000-74633000
					Chr4:9519000-9791000^a^	
Primate	Orangutan	*Pongo pygmaeus*	ponAbe2	v.2.0.2	Chr4:9065000-9474000	Chr17:64862000-65072000
Primate	Macaque	*Macaca mulatta*	rheMac2	v.1	Chr5:4455000-4928000	Chr16:70135000-70333000
Primate	Marmoset	*Callithrix jacchus*	calJac1	v.2.0.2	Ctg_6604:8000-119000	Ctg_751:460000-660000
Primate	Galago	*Otolemur garnettii*	Table 2^c^		Table 2^c^	Table 2^c^
Rodent	Mouse	*Mus musculus*	mm9	v.37	Chr5:38602000-38894000	Chr11:115128000-115293000
Rodent	Rat	*Rattus norvegicus*	rn4	v.3.4	Chr14:77872000-77739000	Chr10:105341000-105511000
Rodent	Guinea pig	*Cavia porcellus*	cavPor3	v.1	Sc_85:1472000-1733000	Sc_76:158000-328000
Odd-toed ungulate	Horse	*Equus caballus*	equCab1	v.1	Chr3:105657000-106026000	Chr11:5605000-5775000
Even-toed ungulate	Cow	*Bos taurus*	bosTau4	v.4.0	ChrUn.004.380:64500-130400	Table 2^c^
Carnivore	Cat	*Felis catus*	felCat3	v.3	Scaffold_217612:1-354000	Sc_213691:103000-208000
Carnivore	Dog	*Canis familiaris*	Table 2^c^		Table 2^c^	Table 2^c^
Insectivore	Hedgehog	*Atelerix albiventris*	Table 2^c^		N/A^d^	Table 2^c^
Dasypodidae	Armadillo	*Dasypus novemcinctus*	Table 2^c^		Table 2^c^	Table 2^c^
**Non-placental mammals**						
Marsupial	Opossum	*Monodelphis domestica*	monDom4	v.1	Chr5:220402000-221007000	Chr2:214376000-214751000
Monotreme	Platypus	*Ornithorhynchus anatinus*	Table 2^c^		Table 2^c^	N/A^d^
**Non-mammals**						
Bird	Chicken	*Gallus gallus*	galGal3	v.2.1	Chr4:81337000-81509000	Chr18:10466000-10598000
Amphibian	Xenopus	*Xenopus tropicalis*	xenTro2	v.4.1	Sc_441:1-372000	Table 2^c^
Reptile	Lizard	*Anolis carolinensis*	AnoCar	v.1.0	Sc_326:248900-318100	Sc_19:2503800-2589100
Teleost fish	Stickleback^b^	*Gasterosteus aculeatus*	gasAcu1	v.1	ChrXVII:8677000-8781000	ChrUn:25945000-25970000^b^
						ChrXI:8905000-10436000^b^
Teleost fish	Zebrafish	*Danio rerio*	Zv8/danRer6		Chr14:404900-441700	Chr3:55047100-55115900 ^b^
						Chr3:58856300-58928900 ^b^
Teleost fish	Medaka	*Oryzias latipes*	oryLat2	v.1.0	Chr5:15877500-15911600	Chr8:10213600-10262900 ^b^
						Chr8:11920390-11941600 ^b^
Teleost fish	Tetraodon	*Tetraodon nigroviridis*	tetNig2	v8	Chr11:1818700-1842400	Chr3:10856000-10898300 ^b^
						Chr3:9834800-9861700 ^b^
Teleost fish	Fugu	*Takifugu rubripes*	JGI 4.0/fr2	v4.0	ChrUn:188417600-188434600	ChrUn:188238700-188275500 ^b^
						ChrUn:273950651-273965439 ^b^

^a^The *Otop1 *locus actually resides in two loci in these hominoid species (with respect to the mouse reference sequence; see text for details).

^b^The *Ush1g-Otop2-Otop3 *locus is duplicated in teleost fish (with respect to the mouse reference sequence; see text for details).

^c^Bacterial Artificial-Chromosome (BAC) clones spanning one or both of the targeted genomic regions were isolated and sequenced (see Table 2 for details and GenBank accession No.).

^d ^N/A, not available: neither BAC- nor whole-genome-derived sequence of the targeted genomic regions could be obtained.

**Table 2:** General features of comparative sequence data set

			**No**.	Clone	Sequence	Total	GenBank
Group	Species	Name	BACs	**Gaps**^**a**^	**Gaps**^**b**^	**Sequence**^**c**^	**Accession No**.
***Otop1 *locus**							
Primate	Galago	*Otolemur garnettii*	4	0	26	763135	DP000201
Carnivore	Dog	*Canis familiaris*	4	1	25	599455	DP000200
Dasypodidae	Armadillo	*Dasypus novemcinctus*	4	2	14	566298	DP000198
Monotreme	Platypus	*Ornithorhynchus anatinus*	1	0	12	115536	DP000202
***Ush1g-Otop2-Otop3 *locus**							
Primate	Galago	*Otolemur garnettii*	3	0	16	415030	DP000189
Carnivore	Dog	*Canis familiaris*	2	0	25	294612	DP000188
Dasypodidae	Armadillo	*Dasypus novemcinctus*	2	0	19	228380	DP000186
Even-toed ungulate	Cow	*Bos taurus*	2	1	11	310444	DP000187
Insectivore	Hedgehog	*Atelerix albiventris*	3	0	16	410814	DP000190
Amphibian	Xenopus	*Xenopus tropicalis*	1	0	12	153069	DP000192

^a^Number of sequence gaps due to the lack of BAC coverage across an interval.

^b ^Number of gaps within the assembled sequences of individual BACs.

^c ^Total non-redundant nucleotides in the assembled sequences of all BACs from that species (As, Gs, Cs, and Ts; not Ns).

### Evolutionary history of the *Otop1* locus in vertebrates

**Figure 1:**
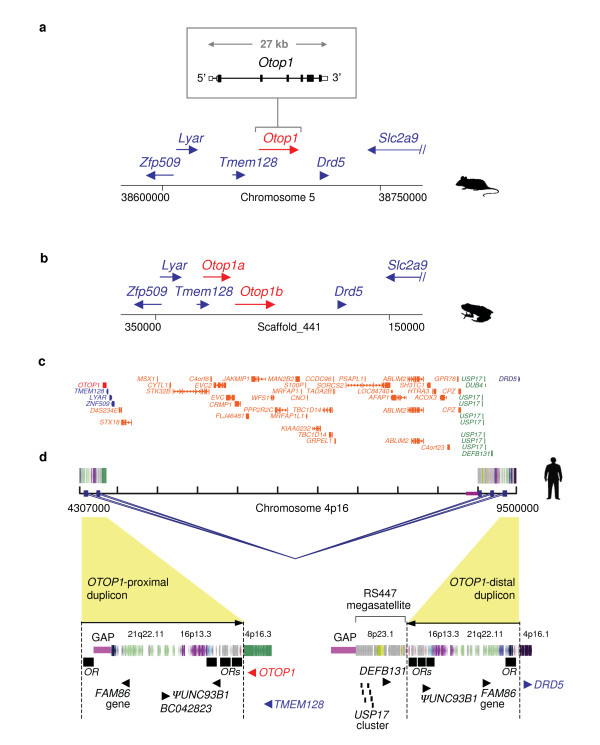
**Genomic architecture of the *Otop1* locus in vertebrates**. **(a) **Mouse genomic region encompassing *Otop1*. The relative positions and transcriptional orientation of the indicated genes are drawn to scale, with the genomic structure of *Otop1 *provided in greater detail. The identical organization of genes within the depicted genomic region is also found in orangutan, macaque, galago, rat, guinea pig, horse, cat, dog, armadillo, opossum, platypus, chicken, and stickleback. **(b) ***X. tropicalis *genomic region encompassing *Otop1 *genes. Note the presence of two paralogous *Otop1 *genes (*Otop1a *and *Otop1b*), but otherwise the same general gene organization as in mouse. **(c) **Human genomic region encompassing *OTOP1 *showing the inversion separating *OTOP1 *and *DRD5*. The ~5-Mb inverted segment is flanked by large, highly similar SDs arranged in a palindromic fashion. The connecting blue lines indicate regions of paralogy between the proximal and distal duplicons (see also additional file 2 for additional pair-wise homology information). The underlying structure of each duplicon-containing region is depicted by colored vertical lines. Selected gene annotations from the UCSC Genome Browser RefSeq track are also shown: *OTOP1 *(red), genes residing in proximity to *Otop1 *in panels **(a) **and **(b) **(blue), genes within the RS447 megasatellite (green), and genes inverted in the human genome compared to species' genomes (orange). **(d) **Fine structure of the SDs flanking *OTOP1 *and the RS447 megasatellite in the human genome. Each duplicon is a mosaic of smaller duplicated segments that are labeled and colored based on their ancestral cytogenetic band of origin. Sequencing gaps in the hg17 sequence assembly of the human genome are indicated with pink lines, while the gene content is annotated below each duplicon. Note the 7E OR clusters residing at each duplicon boundary (black boxes) and palindromic configuration of the proximal and distal duplicons.

In 20 of the 25 vertebrate genomes studied, the immediate genomic context for *Otop1 *is identical (with *Otop1 *flanked by *Tmem128 *and *Drd5 *on the 5' and 3' side, respectively; Figure 1a). For three of the remaining species (cow, marmoset, and hedgehog), there were insufficient sequence data to draw firm conclusions about the genomic context of *Otop1*. In *X. tropicalis*, instead of a single *Otop1 *gene, *Tmem128 *and *Drd5 *flank two closely spaced *Otop1 *genes, which we have named *Otop1a *and *Otop1b *(Figure 1b; and additional file 1).

**Figure 2:**
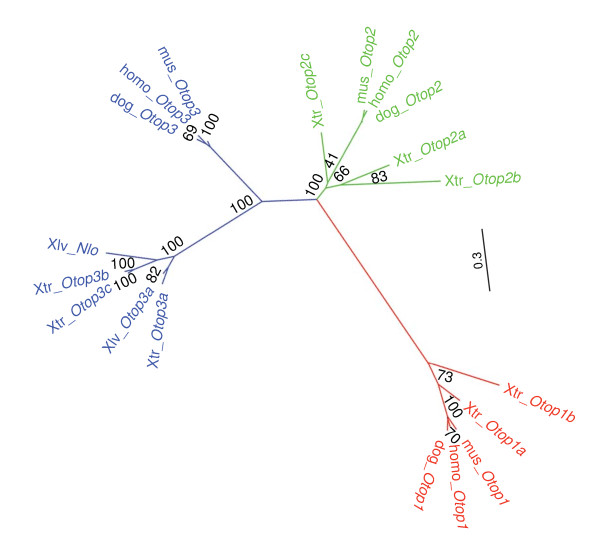
**Phylogeny of the Otop family in amphibians**. Maximum-likelihood phylogenetic tree based on the multi-sequence alignment of 19 Otop proteins identified in amphibian, mouse, human, and dog. Proteins are labeled as Xtr_ for *X*. *tropicalis*, Xlv_ for *X. laevis*, mus_ for mouse, homo_ for human, and dog_ for dog. Three distinct clades divide the Otop family into three subfamilies: Otop1, Otop2, and Otop3 (colored red, green, and blue, respectively). Amphibian *Otop3 *genes appear to have undergone additional gene-duplication events, creating lineage-specific paralogs (designated a to c following the gene symbol). We have applied the same naming convention to the *X. tropicalis Otop1 *(*Otop1a *and *Otop1b*) and *Otop2 *(*Otop2a*, *Otop2b*, and *Otop2c*) genes, although it is less clear if the duplication events giving rise to these multiple copies occurred in the amphibian lineage or are more ancient (with the genes then getting lost in the mammalian lineage). Branch labels are bootstrap values for 1000 replicates.

In our maximum likelihood analysis of the Otop family (Figure 2), the Otop1a-encoding gene resides within a clade containing the mammalian Otop1-encoding genes, while the Otop1b-encoding gene resides within a sister clade. A strict interpretation of this tree (Figure 2) requires the loss of an *Otop1b *gene in the lineage leading to mammals. Having the genomic sequence of the *Otop1*-containing region of *X. laevis *or another amphibian might be useful for timing this genomic event (note that it has not been determined whether the *X. laevis *genome contains multiple *Otop1 *genes). The *X. tropicalis Otop1a *and *Oto1b *genes encode predicted proteins that are 69% identical to each other at the amino-acid sequence level, and 72% and 65% identical to the mouse *Otop1 *protein, respectively. Otop1a more closely resembles mammalian Otop1, suggesting that, upon duplication, Otop1b may have acquired novel functions.

The most dramatic difference in the architecture of the *OTOP1*-containing genomic region is in the human genome, in which *OTOP1 *is the boundary gene of a submicroscopic inversion polymorphism on chromosome 4p16. In the inverted configuration (represented in the hg17 reference sequence), *OTOP*1 and *DRD*5 are separated by ~5 Mb, with the break in synteny (with respect to mouse chromosome 5) occurring immediately adjacent to the 3' end of *OTOP1 *(Figure 1c). All genes in this large genomic segment have the opposite orientation to most other vertebrate genomes studied.

**Figure 3:**
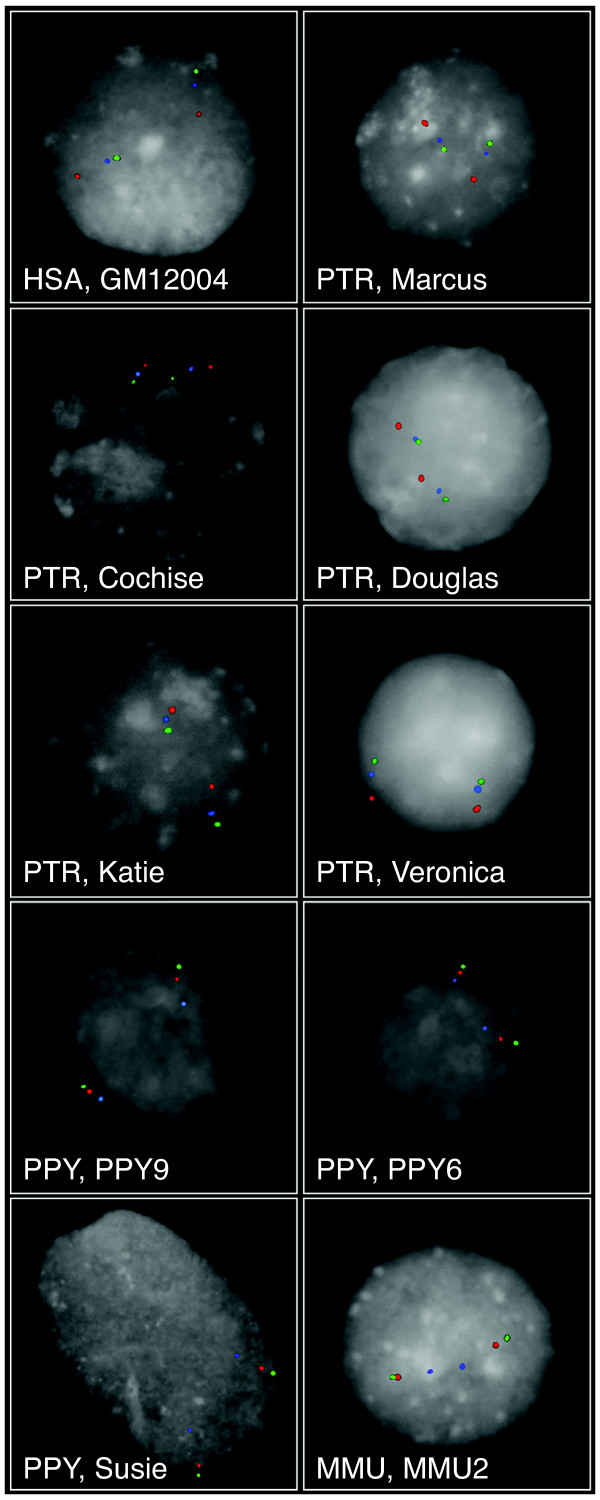
**Inversion analysis of the genomic region encompassing *OTOP1***. Three-color interphase FISH using probes WIBR2-1849E16 (red), WIBR2-1416B12 (blue), and WIBR2-1634L14 (green) was used to determine the orientation of the *OTOP1*-containing region in the human (HSA), chimpanzee (PTR: Marcus, Cochise, Douglas, Katie, and Veronica), orangutan (PPY; PPY9, PPY6, and Susie), and macaque (MMU) genomes. The inversion changes the order of the red and blue probes (mapping inside of the inversion) and their relative position with respect to the green probe (mapping outside of the inversion). FISH results show inversion of the region in human and chimpanzee with respect to orangutan and macaque.

The inversion polymorphism on human chromosome 4p16 was previously observed in a heterozygous state in 12.5% of a Caucasian population [15]. Large (e.g., ~5 Mb) inversions are uncommon in the human genome; in fact, within euchromatin, there are relatively few greater than 1 Mb. We thus sought to clarify the evolutionary origins of this inversion by comparisons with three non-human primate species serving as outgroups. The orientation of the *OTOP1*-containing region was validated by three-color interphase fluorescence in situ hybridization (FISH) [25] studies involving a single human individual, five chimpanzees, three orangutans, and one macaque. To visualize the inversion, we selected two probes inside (red and blue) and one outside (green) the inverted region. The presence of an inversion changes the order of the red and blue probes and their position relative to the green probe (Figure 3). These results demonstrate the inversion of the *OTOP1*-containing region in the human individual and in all chimpanzees sampled, but not in any orangutan or macaque studied, suggesting that the inversion might have occurred in the shared human-chimpanzee lineage and could still reflect a polymorphism in humans. It is not possible to establish the polymorphic or fixed status of this inversion in the chimpanzee lineage because of the small number of chimpanzees studied (five).

### Evolutionary history of the human chromosome 4p16 inversion breakpoints

The polymorphic *OTOP1*-containing inverted segment is flanked by ~260-kb duplicated segments, also referred to as segmental duplications (SD; Figure 1d; and additional file 2). These are highly similar sequences (>95% sequence identity) located near the *OTOP1*-proximal and *OTOP1*-distal inversion breakpoints at positions ~4 Mb and ~9 Mb on human chromosome 4 (referred to as positions Chr4:4 and Chr4:9, respectively, by Darai-Ramqvist *et al*. [26]). The SDs are oriented in an inverted configuration, suggesting that they originated by a non-allelic homologous recombination event. Each SD consists of a mosaic of duplicated sequences originating from multiple chromosomes, most likely compiled in a step-wise fashion [27,28]. The immediate boundary region between *OTOP1 *and the *OTOP1*-proximal duplicon contains one retroposed pseudogene *(ΨUNC93B1*). Both SD boundaries are enriched for olfactory receptor (OR)-gene sequences from the 7E subfamily [29]; specifically, each cluster of 7E OR sequences consists of one or more ~13-kb segment(s) containing a 7E OR gene interspersed with LINEs, SINEs, the LTR-containing satellite repeat SATR, and HERVE retroviruses arranged in a characteristic pattern (Figure 1d; also see [29]).

Of note, the *OTOP1*-distal SD (also flanked by clusters of OR-gene sequences) lies immediately adjacent to the RS447 megasatellite, a tandem repetitive sequence containing copies of the deubiquitinating enzyme gene *USP17 *(Figure 1d). Bioinformatic analyses have revealed the presence of a less-conserved, minor RS447 locus on human chromosome 8p23, which is relevant to our study (as discussed below).

**Figure 4:**
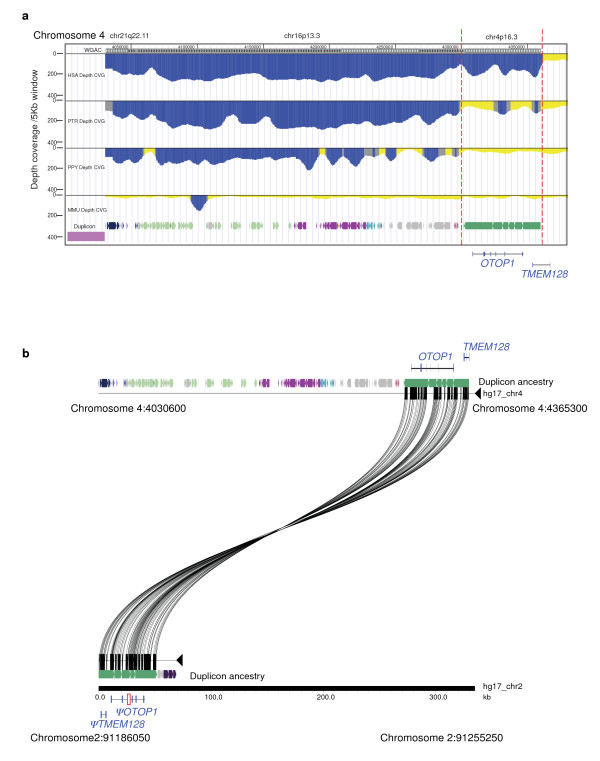
**Evolutionary analysis of the *OTOP1*-proximal locus in the human genome**. **(a) **SDs in human (HSA), chimpanzee (PTR), orangutan (PPY), and macaque (MMU) genomes, as detected by excess of whole-genome shotgun reads (depth of coverage). Note the evidence for a ~60-kb human-specific SD containing *OTOP1*-like and *TMEM128*-like sequences (i.e., the region between the vertical red dashed lines). **(b) **The genomic regions in chromosomes 4 and 2 were extracted from the human assembly hg17 and aligned with Miropeats Pertinent gene annotations and ancestral duplicon composition of each duplicon (obtained from DupMasker) are also shown. Newly identified Ψ*OTOP1 *and Ψ*TMEM128 *belong to a single duplication event located in the pericentromeric region of human chromosome 2. The red box represents an indel that has deleted exon 4 of Ψ*OTOP1*.

We next analyzed a map of known SDs in the primate genome [30,31] to infer the evolutionary history of the *OTOP1*-flanking SD. As depicted in Figure 4a (and expanded in additional file 3), this family of SDs, also called the tumor break-prone segmental duplication (TBSD) family [26], is specific to great apes, emerging and spreading out in hominid genomes since the divergence of the human, chimpanzee, and orangutan common ancestor from the macaque lineage, roughly 12-16 million years ago. All SDs belonging to the TBSD family are >95% identical and have distinctive boundaries enriched in 7E OR genes, LTR-containing retroviruses, and copies of the retroposed pseudogene *ΨUNC93B1*. The phylogenetic relationships among the 7E OR genes have been studied by others [29] revealing unique features, such as a high frequency of gene conversion with distant neighbors and evidence of inter-and intra-chromosomal duplication events. This pattern is distinct from the entire set of human SDs, of which 86% duplicated intra-chromosomally [32]. Collectively, these data support the idea that the highly similar 7E OR gene clusters at the boundaries of TBSD family members may predispose to chromosome breakage and rearrangement; this likely reflects the history of the human chromosome 4p16 inversion described here. Additional details about the TBSD family in primates can be found in additional files 4, 5, and 6.

Our analyses also revealed a second SD (~60 kb in size) in the human genome containing *OTOP1*- and *TMEM128*-like sequences (Ψ*OTOP1 *and Ψ*TEM128*, respectively; Figure 4a and 4b). Here, the boundaries lacked the distinctive features of the TBSD family members. This small duplication most likely resulted from a typical duplication-shadowing event [30,31]. Furthermore, oligonucleotide-based comparative genomic hybridization (array CGH) [30] confirmed that this duplicated segment is present in single copy in human and gorilla genomes but, interestingly, not in the chimpanzee or bonobo genomes (additional file 3). This duplication pattern is inconsistent with the generally accepted human-great ape phylogeny; such a scenario may have arisen from a deletion event in the chimpanzee lineage, incomplete lineage sorting or, less likely, a recurrent duplication event in the human and gorilla lineages [30]. In the human genome, this 60-kb duplicon resides in the pericentromeric region of chromosome 2 (Figure 4b). Human Ψ*OTOP1 *is 96% identical to *OTOP1 *at the sequence level, but contains multiple stop codons and indels (e.g., complete deletion of exon 4); it is thus unlikely to encode a functional protein. However, the promoter associated with Ψ*OTOP1 *may still be active, as multiple truncated Ψ*OTOP1 *ESTs exist in GenBank (grouped within UniGene Hs.615109).

### Evolutionary history of the *Ush1g*-*Otop2*-*Otop3* locus in vertebrates

**Figure 5:**
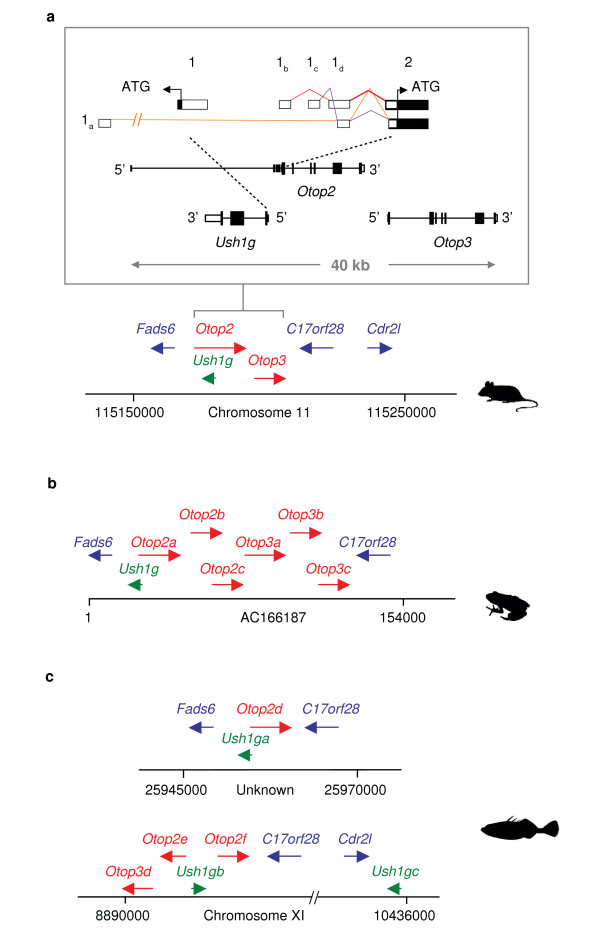
**Genomic architecture of the *Ush1g*-*Otop2*-*Otop3* locus in vertebrates**. **(a) **Mouse genomic region encompassing the *Ush1g*, *Otop2*, and *Otop3 *genes. The relative positions and transcriptional orientation of the indicated genes are drawn to scale, with the genomic structures of *Ush1g*, *Otop2*, and *Otop3 *provided in greater detail. Note the red, orange, and purple lines that indicate how three known splice forms of mouse *Otop2 *are derived from the use of four alternate non-coding exons (named 1_a _to 1_d_) and two internal splice donor sites in exons 1_d _and 2. The identical organization of genes within the depicted genomic region is also in found human, chimpanzee, orangutan, macaque, marmoset, galago, rat, guinea pig, horse, cow, cat, dog, armadillo, opossum, and chicken. **(b) ***X. tropicalis *genomic region encompassing the *Ush1g*, *Otop2*, and *Otop3 *genes. Note the presence of three paralogous genes for both *Otop2 *and *Otop3 *(for details about the phylogenetic relationships of the *Otop *genes in amphibian and selected vertebrates, see Figure 2). **(c**) Stickleback genomic regions containing the *Ush1g*, *Otop2*, and *Otop3 *genes showing a complex duplication and rearrangement pattern (see text for details).

The *Ush1g-Otop2-Otop3 *locus spans ~40 kb in the mouse genome (Figure 5a). The tandem genes *Otop2 *and *Otop3 *have the same orientation and are separated by a 2.4-kb intergenic region, while *Ush1g *has the opposite transcriptional orientation. Based on analyses of mouse ESTs, *Otop2*-derived transcripts yield three alternatively spliced variants derived from the use of four non-coding first exons (1_a _to 1_d_) as well as two internal splice sites in exon 1_d _and the non-coding portion of exon 2 (Figure 5a inset). The presence of exon 1_a_, 1_b_, or 1_c _in an *Otop2*-derived transcript is mutually exclusive of the others, while all transcripts share parts of exon 1_d _and the non-coding portion of exon 2. Consistent with its presence in all *Otop2*-derived splice forms, exon 1_d _is highly conserved among vertebrates (e.g., mouse and opossum exon 1_d _are 60% identical at the nucleotide level, with highly conserved canonic donor and acceptor splice sites), and the exon's internal acceptor splice site is conserved across rodents, primates, carnivores, and ungulates. Exon 1_c _is well conserved among vertebrates, with the exception of mouse and rat, which have divergent sequences but a conserved splice-donor site. Finally, exons 1_a _and 1_b _show neither sequence nor donor splice-site conservation among vertebrates except in mouse and rat, suggesting that these exons may be rodent-specific. Thus, *Ush1g *is not adjacent to, but embedded within, the first intron of *Otop2 *in rodent genomes due to the presence of lineage-specific *Otop2 *untranslated exons.

An identical repertoire of genes near the *Ush1g-Otop2-Otop3 *locus is present in 18 of the 25 genomes studied, including those encoding: (1) fatty acid desaturase domain family member 6 (*Fads6*) nearest the 5' end of *Otop2; *and (2) hypothetical protein (*C17orf2*) and cerebellar degeneration-related protein 2-like (*Cdr21*) nearest the 3' end of *Otop3 *(Figure 5a). The exceptions are platypus (for which we were unable to generate or recover a sequence for this genomic region); and *Xenopus *and the five fish lineages (all of which have notably different structures for this genomic region).

The *Ush1g-Otop2-Otop3 *locus is markedly different in frog and fish lineages compared to the other vertebrates studied. Specifically, the *X. tropicalis *genome contains only one copy of the *Ush1g *gene (similar to other vertebrate genomes), but *Otop*2 and *Otop3 *are uniquely expanded into a cluster of six paralogous genes (Figure 5b). A maximum likelihood (ML) unrooted tree that includes all known amphibian, mouse, human, and dog Otop sequences is shown in Figure 2. Three well-supported clades representing the Otop1, Otop2, and Otop3 subfamilies (labeled in red, green, and blue, respectively) are seen, indicating that the genome of the last common ancestor of placental mammals and amphibians (~320-360 million years ago) had at least three *Otop *paralogs.

Determining the timing of the *Otop *duplication events is difficult. The tree in Figure 2 suggests that the ancestry of Otop1- and Otop2-encoding genes may have been shaped by a combination of ancient duplication events and mammalian-specific gene loss. Alternatively, our tree may be thrown off by to the asymmetric evolutionary rates of daughter genes following duplication and divergence--with the duplicate that retains the ancestral function evolving more slowly than the other(s) (see discussion in Larroux *et al.*, [33]). Including additional *X. laevis Otop1 *and *Otop2 *sequences (if they exist and are found in the future) may help break longer branches and clarify these relationships. On the other hand, the *Otop3 *scenario seems to be more clearly a result of amphibian-specific duplication. Furthermore, the topology of the two X. *laevis Otop3*-like sequences (*Nlo *[20] and this study) suggests that at least one of the *Otop3 *duplication events occurred prior to the last common ancestor of *X. laevis *and *X. tropicalis*. Notably, *Nlo *is expressed within the non-neural ectoderm surrounding the anterior neural plate of *X. laevis *embryos; at tailbud stages, *Nlo *is expressed in the dorsolateral region of the otic vesicle, which later gives rise to the gravity organs. Given the phylogenetic relationship of *Nlo *and *Otop3 *as well as the apparent role of amphibian *Nlo*, mouse *Otop1*, and zebrafish *Otop1 *in the development of the vestibular system, it seems possible that the ancestral *Otop *gene was involved in vestibular system development.

We examined whole-genome sequence assemblies for five fish species (zebrafish, *Danio rerio*; tetraodon, *Tetraodon nigroviridis*; fugu, *Takifugu rubripes*; stickleback, *Gasterosteus aculeatus*; and medaka, *Oryzias latipes*). We emphasize our analyses of the stickleback genome in Figure 5c because its whole-genome sequence assembly included the best coverage of the *Ush1g-Otop2-Otop3 *locus among fish. Consistent with the whole-genome duplication that occurred in teleost fish lineage subsequent to its divergence from mammalian ancestors ~230 million years ago [34], the *Ush1g-Otop2-Otop3 *locus occupies two locations in the stickleback genome. One segment (not assigned to a chromosome, ChrUn: 25945000-25970000) shows conserved synteny with other vertebrate genomes, with *Fads6*, *Ush1ga*, *Otop2*, and *C17orf28 *similarly ordered and oriented (but not *Otop3*; see Figure 5c). The other segment, residing on chromosome XI, appears to result from duplication and rearrangement events, yielding broken synteny among the genes (Figure 5c); this region is not fully characterized, but in the stickleback whole genome assembly it contains at least two *Otop2*-related genes (*Otop2d *and *Otop2e*), one *Otop3*-related gene (*Otop3d*), and two copies of *Ush1g *(*Ush1gb *and *Ush1gc*). Note that *Ush1ga *and *Ush1gb *(but not *Ush1gc*) have remained close to *Otop*-related sequences. A total of 15 *Ushg1 *genes were annotated in the whole-genome sequence assemblies of the five fish (for the complete listing and coordinates of all fish *Ushg1 *genes see additional files 7, 8, 9, 10 and 11). Multi-species comparisons of the predicted protein sequences revealed that the defining architectural features of Ush1g in placental mammals (i.e., three ankyrin domains and a SAM domain) are highly conserved in the three fish Ush1g paralogs, with most sequence variation residing within the central region of the protein of unknown function (additional file 12).

### Conserved non-coding sequences within the *Ush1g*-*Otop2*-*Otop3* locus

We next set out to identify conserved non-coding sequences of potential functional relevance within the ~40-kb *Ush1g*-*Otop2-Otop3 *locus. We analyzed the high-quality sequences generated from galago, dog, armadillo, cow, and hedgehog in conjunction with the orthologous mouse and human sequences from the UCSC Genome Browser. The *X. tropicalis *sequence was not included in this analysis because its unique *Otop *family expansion complicates the alignment of non-coding sequences with other species' sequences. ExactPlus [35] was used to identify multi-species conserved sequences (MCSs) within the multi-sequence alignments. MCSs overlapping known coding sequences were removed, thereby enriching conserved non-coding sequences. The resulting set of 67 non-coding MCSs (from 7-31 bp long and averaging 12 bp) together span 806 bp (2%) of the analyzed interval.

To assess ExactPlus' performance, we compared its output with data from the "PhastCons Conserved Elements, 30-way Vertebrate Multiz Alignment" track on the UCSC Human Genome Browser; this track depicts the top 5% most-conserved MCSs based on PhastCons analyses of 30 vertebrate genome sequences [36]. Roughly 350 bp of non-coding sequence were identified by both ExactPlus and PhastCons, accounting for ~0.8% of the analyzed ~40-kb interval. The first intron of *USH1G*, in particular, appears to contain a set of regulatory sequences with characteristics that are well conserved among vertebrates: a uniform intron size of ~2.2 kb; virtual absence of repetitive elements across species; and richer in highly conserved non-coding elements than the surrounding genomic regions (see additional file 13 for the complete listing and coordinates of non-coding MCSs identified by ExactPlus and PhastCons).

**Figure 6:**
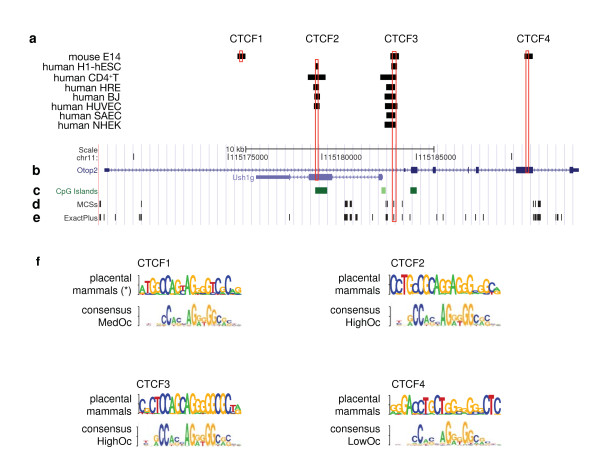
**CTCF-binding sites within the *Ushg1*-*Otop2* locus**. The coordinates of the depicted genomic region in the mouse genome (assembly NCBI/mm9) are Chr11:115168200-115193650. (**a**) Position of mapped sequence reads from ChIP-seq studies using an anti-CTCF antibody and the following cells: mouse E14 ES cell line [37]; human H1-hESC ES cell line (Duke/UNC/UT-Austin/EBI ENCODE group; http://genome-test.cse.ucsc.edu); resting human CD4+T cells [38]; and five non-cancerous, karyotypically normal human cell lines (HRE, human renal epithelial cells; BJ, skin fibroblasts; HUVEC, human umbilical vein endothelial cells; SAEC, small airway epithelial cells; and NHEK, normal human epidermal keratinocytes [Duke/UNC/UT-Austin/EBI ENCODE group]). For display purposes, the coordinates of the human CTCF-binding fragments CTCF2 and CTCF3 are presented based on the coordinates in the mouse genome. Based on these data, four CTCF-binding sites (CTCF1 to CTCF4) were identified with the following species-specific occupancy: CTCF1 and CTCF4, mouse-specific; CTCF2, human-specific; and CTCF3, both mouse and human. The mouse *Ushg1 *and *Otop2 *gene structures (**b**) and CpG-island content (**c**) were derived from the RefSeq and CpG island tracks of the UCSC Genome Browser (blue and green boxes, respectively). Black boxes represent non-coding MCSs identified by both ExactPlus and PhastaCons (**d**) or ExactPlus only (**e**), respectively. The open red boxes highlight the position of the CTCF-binding motifs. (**f**) Sequence Logos for CTCF1 to CTCF4 graphically represent the multi-sequence alignment at the CTCF-binding sites in placental mammals; the height of each symbol reflects the relative frequency of that nucleotide at that position. (*) Indicates that hominoid sequences were not considered for the Logo generation of CTCF1 due to lack of motif conservation (i.e., hominoid-specific deletion of base 9). Consensus Logo motifs for low-, medium-, and high-occupancy CTCF-binding sites (LowOc, MedOc and HighOc, respectively) are also shown; these classes are based on the degree to which the CTCF-binding sites match the known CTCF-binding motif and the densities of sequence reads mapped at the binding sites [40,41].

An open question is whether there are non-coding sequences in and around the *Ushg1*-*Otop2 *locus that act as 'functional boundaries'- either to insulate these genes from each other or to orchestrate their regulation. Chromatin immunoprecipitation-sequencing (ChIP-seq) combines chromatin immunoprecipitation of a DNA-binding protein with high-throughput DNA sequencing and mapping of the 'sequence tags' to a reference genome. An obvious candidate for organizing chromatin domains is the mammalian insulator CTCF. We thus mined data from *in vivo *genome-wide ChIP-seq studies in human and mouse ES cells for CTCF 'sequence tags' that map to the *Ush1g-Otop2 *locus (Duke/UNC/UT-Austin/EBI ENCODE group [http://genome-test.cse.ucsc.edu] and Chen *et al.*, [37], respectively). Four putative CTCF-binding sites (Figure 6a) were found with the following species-specific occupancies: CTCF1 (located at the 3' end of *Ush1g*) and CTCF4 (within exon6 of *Otop2*), mouse-specific; CTCF2 (within exon 2 of *Ush1g*) human-specific; and CTCF3 (in between *Ushg1 *and *Otop2) *occupied in mouse and human ES cells. Notably, the binding of CTCF to CTCF3 has been replicated in all available genome-wide ChIP-Seq studies, including resting human CD4+T cells [38], five differentiated non-cancerous, karyotypically normal human cell lines (Figure 6a), and a number of immortalized human cancer cell lines ([39] and http://genome-test.cse.ucsc.edu; data not shown).

*In silico *analyses confirmed consensus CTCF-binding motifs within the sequence of all four CTCF-binding locations (Figure 6f) [40]. Typical CTCF-binding sites are 20 bp long, with two conserved cores: one spanning bases 4-8 and the other bases 10-18 (Figure 6f). Furthermore, CTCF-binding sites can be grouped into low-, medium-, and high-occupancy sites (LowOc, MedOc, and HighOc, respectively) depending upon how closely they match the consensus CTCF-binding motif and the reported density of mapped tags. Each class has statistically divergent features, suggesting distinct functional roles [41]. CTCF3 contains a HighOc binding site that is highly conserved across placental mammals. Statistically, HighOc sites tend to be more conserved than their flanking regions, are ubiquitously bound by CTCF, and act as chromatin barriers less often than LowOc sites; however, paradoxically, they have a greater tendency to delimit domains of co-regulated genes [41].On the other hand, CTCF2 (not occupied in mouse ES cells and some differentiated human cell lines) contains a LowOc motif. LowOc binding motifs demonstrate greater conservation in their flanking regions compared to HighOc sites, and tend to be cell-specific, with CTCF-binding being more variable between cell types than at HighOc sites. LowOc sites have been proposed to play a role in establishing chromatin barriers. Neither CTCF2- nor CTCF3-binding motifs are conserved in non-placental vertebrates (data not shown).

CTCF1 and CTCF4 are only occupied in mouse ES cells. Of note, the spacing of the cores within the CTCF1 motif was uniquely lost in human, chimpanzee, and gorilla due to the deletion of spacer base 9; otherwise the binding motif is conserved in placental mammals. Also, CTCF4 contains a strong CTCF-binding site (5' -GAGACCTGCAGGGGGCGCTC) in the mouse genome, although the binding motif is biased towards less-commonly reported bases (e.g., an almost fixed T in position 10, instead of an A) in other placental mammals.

## Discussion

In this study, we used comparative genome sequencing and cytogenetics to examine the evolutionary history and the genomic context of three *Otop *genes in 25 evolutionarily diverse vertebrate species. We also extended our evolutionary studies to the *Ush1g *deafness gene because of the tight head-to-tail physical clustering of *Ush1g *with *Otop2 *and *Otop3 *in vertebrate genomes, and because mutations in *Otop1 *and *Ush1g *result in inner ear phenotypes in vertebrates. Based on our analyses, we conclude that the evolution of the *Otop *family in hominoids, amphibians, and rodents significantly departs from that of most vertebrate genomes, as does the evolution of *Ush1g *in the teleostei fish lineages.

The most striking difference found between hominoid species and other vertebrates is that the *OTOP1 *locus is flanked by a large SD of high complexity and sequence identity, belonging to the TBSD family, and arranged in an inverted orientation. Furthermore, *OTOP1 *sits only 3 kb away from the *OTOP1*-proximal inversion boundary. Thus, the immediate genomic context of human *OTOP1 *differs significantly from that of mouse and zebrafish, the only other vertebrates in which *Otop1 *has been closely studied [4,10]. Therefore, information on regulation of mouse and zebrafish *Otop1 *may not accurately reflect human *OTOP1 *regulation, and/or Otop1 developmental and biochemical function(s) in mouse and fish may be represented by another *OTOP *gene in humans. Using cytogenetic and comparative genomic approaches, we examined the evolutionary history of the hominoid *Otop1 *locus, including the flanking TBSD duplicons. Our findings indicate that the TBSD family emerged at some point after the divergence of the human, chimpanzee, and orangutan common ancestor from the macaque lineage ~12-16 million years ago, and later underwent significant expansion (perhaps within the common ancestor of the great apes). Further, these SDs contribute to plasticity and instability in multiple regions of the genome [26].On human chromosome 4p16 we describe a large (~5 Mb) inversion polymorphism flanked by palindromic TBSD sequences with *OTOP1 *as the boundary gene; this polymorphic arrangement, occurring in one in eight individuals of the Caucasian population, likely originated in the common human-chimpanzee lineage prior to ~6 million years ago.

Our studies, combined with others [42], have yielded a predicted timeline of genomic events affecting human chromosome loci 4p16 and 8p23 that have contributed to the structural complexity and genomic instability of the *OTOP1 *locus in humans; this timeline highlights interesting evolutionary parallels between these two regions. The macaque and mouse genomes are orthologous across the *Otop1 *locus, meaning that *Otop1 *and *Drd5 *are tightly linked, with no evidence for the presence of 7E OR gene clusters, complex SDs, or RS447 microsatellite sequences. RS447 sequences are, however, present in the macaque region orthologous to human chromosome 8p23. In orangutan, clusters of 7E OR and RS447 sequences reside close to each other in both regions, yet there is no evidence for complex mosaic duplications or inversions in either location. Chimpanzee (and bonobo) show increased copy number of the RS447 megasatellite on chromosome 4p16 (as deduced by array CGH; see additional file 3) and evidence for significant expansion of the TBSD family across a number of chromosomes. Furthermore, inversion polymorphisms of similar size (~5 Mb) have been reported on chromosomes 8p23 and 4p16 in humans and chimpanzees.

The mechanism underlying such genomic rearrangements is not completely clear. One possible scenario is that flanking clusters of SDs triggered the inversions via a non-allelic homologous recombination event ([42]; and present study). Alternatively, rather than rearrangements being mediated by the duplications themselves, the inversions could have helped to create the complex segmental duplication architecture present at their breakpoints [43]. Whatever the causal mechanism, the frequency of 8p23 and 4p16 inversion polymorphisms is relatively high in the human population, oftentimes with serious consequences for double heterozygotes. Specifically, mothers who are double heterozygotes for the inversion polymorphisms on chromosomes 4p16 and 8p23 are prone to recurrent de novo t(4;8)(p16;p23) translocations through unusual meiotic exchanges, resulting in offspring with Wolf-Hirschhorn syndrome. The phenotype of patients with this syndrome consists of mental retardation and an array of developmental defects, often including hypotonia (decreased muscle tone; [15,44,45]). It is conceivable that individuals broadly labeled hypotonic might also suffer from vestibular dysfunction, which could go undiagnosed because it is often not clinically assessed. It would thus be interesting to study Wolf-Hirschhorn syndrome patients with balanced t(4;8)(p16;p23) translocations in search of individuals with disrupted or dysregulated *OTOP1 *(via *OTOP1 *copy number changes, formation of *OTOP1 *chimeras, or altered *OTOP1 *position). The resulting physiological consequences could include complete abrogation of OTOP1 function, haploinsufficiency, elevated *OTOP1 *expression, or creation of a fusion protein with a dominant-negative effect or novel gain of function [46], any of which would contribute to our understanding of the role of *OTOP1 *in human development.

*X. tropicalis *is the other vertebrate in which we found significant differences in genomic organization of the *Otop *family. Specifically, the *X. tropicalis *genome contains multiple paralogs for each *Otop *gene. We have determined that amphibian *Nlo *has a phylogenetic relationship to *Otop3*-like genes. Both *Nlo *and *Otop1 *are apparently involved in the development of the vestibular system in amphibians and placental mammals, respectively, suggesting that multiple *Otop *genes may have roles in vestibular system development. Studying the degree of sub-functionalization of *Otop *paralogs in *X. tropicalis *may help to define the unique functions attributable to each paralog.

Amphibians and other vertebrates share similar auditory and vestibular physiology, with their vestibular organs particularly well conserved in position, structure, and function [47]. However, a striking difference is the unusual calcium carbonate crystalline forms of amphibian otoconia. The general trend during vertebrate evolution has been a replacement of otoconial vaterite and aragonite crystals by calcite, a calcium carbonate crystal polymorph of increased stability [48]. Such a trend correlates with the transition of vertebrates from aquatic to terrestrial life. Chondrostei fish have vaterite and/or aragonite crystals, teleostean fish only have aragonitic otoliths, and both mammalian and avian otoconia consist exclusively of calcite crystals. During vertebrate evolution, calcitic otoconia appeared for the first time in the amphibian inner ear, yielding an intriguing intermediate situation with two crystalline forms: calcite in the utricle and aragonite in the saccule, lagena, and endolymphatic sac [49-52].

The structural variation of otoconia among vertebrates is thought to result from different properties of their scaffold proteins. However, the main scaffold protein in amphibians, otoconin-22 (Oc-22), is produced in most portions of the developing inner ear (i.e., saccule, crista ampularis, endolymphatic sac, and utricule [51]), making it unclear how two entirely different crystalline forms of otoconia are regionally specified during amphibian development. Therefore, additional factors unique to either the utricule (with calcitic otoconia) or saccule (with aragonitic otoconia) likely lead to distinct regional nucleation and crystal growth during otoconial formation in amphibians. Reverse genetic screens that might clarify the peculiarities of otoconial formation in amphibians have been impossible to undertake because animal models with clean, non-syndromic balance phenotypes are generally very rare [4], to the point that frog mutants with complete otoconial agenesis have yet to be described. In this regard, a candidate gene approach involving targeted disruption of *Otop *genes in *X. tropicalis*, individually or in combinations, could help discern whether different *Otop *paralogs have acquired discrete functions contributing to the regional differences in otoconial formation; such a study could be performed using morpholinos, which have been successfully used to specifically examine the role of *Otop1 *in vestibular system development in zebrafish [7] and general otic vesicle development in *Xenopus *[53].

Of the 16,000 deaf-blind persons in the United States, more than half are believed to have Usher syndrome (USH), a combination of progressive retinopathy and congenital hearing loss. USH type I is especially devastating because of its early onset and extreme phenotype of profound congenital deafness with unintelligible speech, retinitis pigmentosa within the first decade of life, and vestibular dysfunction*. USHIG *is one of five USH type 1 causative genes identified to date. Those include the genes encoding the molecular motor myosin VIIa (*MYO7A*; USH1B); two cell adhesion cadherin proteins, cadherin 23 (*CDH23*; USH1D) and protocadherin 15 (*PCDH15*; USH1F); and two scaffold proteins, harmonin (*harmonin; *USH1C) and USH1G (*USH1G*; USH1G). These proteins are involved in a protein network or "interactome" within sensory cells of the eye and inner ear [54].

Our evolutionary studies of *Ush1g *in vertebrates revealed a unique expansion of this gene family to include three members in all five fish species studied. A number of zebrafish models of USH have been described, including *mariner *(*Myo7A*, [55]), *sputnik *(*Cdh23*, [56]), and *orbiter (Pcdh15a*, [57]). Our efforts have revealed that multiple copies of *Myo7A, Cdh23, Pcdh15*, and *Ush1g *are present in fish genomes (two, two, two, and three copies, respectively; data not shown). Interestingly, there is evidence that the "USH interactome" may be more complex in fish than in other vertebrates [57]. Specifically, the two *Pcdh15 *orthologs in zebrafish have distinct functions in hearing and vision: *Pcdh15a *is required for normal auditory and vestibular function, while *Pcdh15b *is required for normal photoreceptor outer segment organization and retinal function. Because Ush1g is a scaffold protein, the presence of three *Ush1g *genes in fish may affect the fish "USH interactome" (and consequently eye and inner-ear development) differently than in humans; such differences should be considered when using fish systems as general models for vertebrate ear physiology.

Functional studies that shed light on the cis-regulatory elements and fine genomic architecture of the *Ush1g*-*Otop2 *locus are needed to establish the mechanisms underlying the lineage-specific differences in *Ush1g *function. For instance, like human *USH1g *patients, the *Ush1g*-defective mouse model *Js *is profoundly deaf with vestibular dysfunction; however, unlike human *USH1g *patients, it does not have abnormal retinal phenotype [58]. It is notable that the transcriptional boundaries between *Ush1g *and *Otop2 *are indistinct in rodent genomes due the presence of two rodent-specific *Otop2*, 5' untranslated exons that cause *Ush1g *to overlap with (or be embedded within) the *Otop2 *transcriptional unit; the effect of this configuration on the expression of these two genes is not known.

CTCF is a zinc-finger transcriptional repressor that serves an insulator function to limit the spread of heterochromatin; it can also operate as a transcriptional activator, regulate nuclear localization, and participate in the control of imprinting [59]. The first intron of *Ush1g *contains an extensive number of evolutionarily conserved sequences and is bracketed by two putative CTCF-binding sites, CTCF2 and CTCF3. As a caveat, the predicted CTCF binding sites are based on positional information of sequence reads in chIP-seq studies and need to be further validated. Nonetheless, we suggest that the HighOc CTCF3 site between *Ush1g *and *Otop2 *may be ubiquitously bound by CTCF in mouse and human, while CTCF1, CTCF2, and CTCF4 may participate in gene expression regulation in a cell- and/or species-specific fashion. Further functional characterization of CTCF1 to CTCF4 will be pivotal for determining whether *Ush1g *and *Otop *are functionally insulated or participate in common developmental pathways.

## Conclusions

Distinct evolutionary events have affected the *Otop *and *Ush1g *genes in vertebrate genomes, particularly in humans and commonly used animal models (specifically, mouse, fish, and *Xenopus*). The lineage-specific evolutionary history of these genes may therefore limit the functional information that can be inferred from animal studies. In this regard, our findings should help guide the choice of which animal system to use when investigating *Otop *and *Ush1g *function. For example, sub-functionalization of the *Otop *paralogs in amphibians may make *X. tropicalis *an appropriate model for exploring the organization, function, and regulation of *Otop *genes and their role in inner ear development in vertebrates; and fish may be an effective vertebrate model for discriminating the different functions of *Ush1g*, perhaps offering the ability to define the unique functions in inner ear and eye development attributable to each paralog. We also suggest that humans with chromosome 4p16 rearrangements should be studied for altered *OTOP1 *function, which may clarify this gene's role in human development.

This study also establishes a framework for defining whether and how *Ush1g *and *Otop *participate in common developmental pathways. Future studies will focus on functional validation of the highly conserved non-coding sequences that were identified in the *Ush1g-Otop *locus, including four putative CTCF-binding sites that likely play a role in orchestrating gene expression at this locus.

## Methods

### Generation of comparative sequence data sets

We assimilated a 25-species comparative sequence data set for studying the *Otop1 *and *Ush1g-Otop2-Otop3 *loci (species are listed in Table 1). For seven species, we generated the sequence by targeted mapping and sequencing. Briefly, BAC clones were isolated from the following libraries (see http://bacpac.chori.org for details), as described [22,23]: galago (*Otolemur garnettii*; CHORI-256), dog (*Canis familiaris*; RPCI-81), cow (*Bos taurus*; CHORI-240), armadillo (*Dasypus novemcinctus*; VMRC-5), hedgehog (*Atelerix albiventris*; LB-4), frog (*Xenopus tropicalis*; CHORI-216), and platypus (*Ornithorhynchus anatinus*; CHORI-236). Each library was screened using pooled sets of oligonucleotide-based hybridization probes designed from the established reference sequence of the genomic regions encompassing the mouse *Otop1 *or *Ush1g*-*Otop2-Otop3 *loci (NCBI37, Chr5: 38602000-38894000 and Chr11: 115128000-115293000, respectively). After isolation and mapping, 25 BACs were selected and subjected to shotgun sequencing and sequence finishing, as previously described [24]. Ultimately, we generated the orthologous sequence of the (1) *Otop1 *locus in galago, dog, armadillo, and platypus; and the (2) *Ush1g-Otop2-Otop3 *locus in galago, dog, armadillo, cow, hedgehog, and frog (see Table 2). For each species and locus, a single non-redundant sequence was generated with the individual BAC sequences (i.e., a multi-BAC sequence assembly) using the program TPF Processor (http://www.ncbi.nlm.nih.gov/projects/zoo_seq). The resulting assemblies were manually verified and submitted to GenBank ([GenBank: DP000186-DP000190], [GenBank: DP000192], [GenBank: DP000198], and [GenBank: DP000200-DP000202]; Table 2).

For the remaining species, and to capture data not derived from our targeted sequencing efforts, we retrieved the orthologous sequences from whole-genome sequence assemblies available on the UCSC Genome Browser ([60]; see genome.ucsc.edu). Identifying the sequences of interest within these assemblies was generally straightforward. One exception involved the detection of a transcribed pseudogene (Ψ*OTOP1*) located in the pericentromeric region of human chromosome 2 (NCBI human genome sequence build 35, Chr2:91186050-91237900); this region was not included in our data set and was identified by BLAST [61] analysis using *OTOP1 *as a query. Our collective efforts yielded the sequences of both genomic regions from nearly all of the 25 vertebrates listed in Table 1. In addition to the data reported here, 22 additional low-coverage (~2-fold redundancy) vertebrate whole-genome sequence assemblies were examined. At the level of resolution offered by these sequences, no new instances of lineage-specific evolutionary events affecting the *Otop *and *Ush1g *families were detected (data not shown).

#### Sequence annotations, alignments, and comparisons

The assembled BAC sequences were annotated for gene content based on alignments to human RefSeq mRNA (or species-specific mRNA, if available) sequences using Spidey (http://www.ncbi.nlm.nih.gov/spidey) and BLAST [61]. Known repetitive sequences were detected by RepeatMasker (http://www.repeatmasker.org) using appropriate repeat libraries for each species. In addition, Sequin (http://www.ncbi.nlm.nih.gov/Sequin) was used to import and confirm all annotations, including verifying splice-site consensus sequences, exon structure, and predicted protein sequences. Pair-wise and multi-species sequence comparisons were performed using MultiPipMaker [62], first using the mouse sequence and then the human sequence as the reference. Multi-species alignments of nucleotide or deduced protein sequences were refined with Sequence Alignment Editor (Se-Al, http://evolve.zoo.ox.ac.uk). Sequence Logos for CTCF1 to CTCF4 were based on multi-species sequence alignments in placental mammals, as prepared with WebLogo v2.8.2 (http://weblogo.berkeley.edu/). All data associated with these analyses are available in GenBank or in the form of UCSC custom track bed files (see additional files 1, 7, 8, 9, 10 and 11).

The program Miropeats [63] was used to align genome sequence assemblies, to determine the length, location, and relative orientations of the segmental duplications (see hg17 *versus *hg19, additional file 5; and *OTOP1*-containing region in Chr4 *versus *Ψ*OTOP1*-containing region in Chr2, Figure 4b), and to display such DNA sequence similarity information graphically. The order and orientation of mosaic duplicons within complex duplication blocks was delineated with DupMasker [28] with customized Perl scripts.

#### Tri-color interphase FISH analyses

Interphase nuclei were prepared from lymphoblast cell lines from human and three primate outgroup species as follows: human cell line GM12004 (Coriell Cell Repository, Camden, NJ); chimpanzees Marcus, Cochise, Douglas, Katie, and Veronica (unknown source; samples donated by Dr. Mariano Rocchi and Dr. Mario Ventura); macaque MMU2 (*Macaca mulatta; *sample donated by Dr. Mariano Rocchi and Dr. Mario Ventura); and orangutan cell line pr01109 (Susie; Coriell Cell Repository, Camden, NJ) and individuals PPY9 and PPY6 (unknown source; samples donated by Dr. Mariano Rocchi and Dr. Mario Ventura). FISH analyses were performed using fosmids WIBR2-1849E16, WIBR2-1416B12, and WIBR2-1634L14, which were labeled by nick-translation with Cy3-dUTP (Perkin-Elmer), Cy5-dUTP (Perkin-Elmer), and fluorescein-dUTP (Enzo), respectively, as generally described by Lichter *et al*. [25]. Briefly, 300 ng of labeled probe were used; hybridizations were performed at 37°C in 2XSSC, 50% (v/v) formamide, 10% (w/v) dextran sulphate, and 3 μg sonicated salmon sperm DNA in a volume of 10 μl; posthybridization washing was performed at 60°C in 0.1XSSC (three times, high stringency); and nuclei were simultaneously DAPI stained. Digital images were obtained using a Leica DMRXA2 epifluorescence microscope equipped with a cooled CCD camera (Princeton Instruments). DAPI, Cy3, Cy5, and fluorescein fluorescence signals (each detected with specific filters) were recorded separately as gray-scale images. Pseudocoloring and merging of images were performed using Adobe Photoshop software. A minimum of 50 interphase cells were analyzed for each hybridization.

#### Detection of SDs

SDs in genomic regions of interest were identified by two analytical approaches: assembly-dependent (whole-genome assembly comparison, or WGAC) and assembly-independent (whole-genome shotgun sequence detection or WSSD) [64]. WGAC is a self-self BLAST-based strategy optimized to provide pair-wise relationships among the SDs within one species. WSSD detects SDs as sequences with overrepresented depth of coverage in randomly selected sequences from a human and non-human whole-genome sequence assembly, using the human sequence assembly as a reference. Inferences can thus be made about the duplication status in different primates [30]. The duplicon content analysis was based on the study by Jiang *et al*. [28], in which the evolutionary history of every duplicon of the human genome was reconstructed with a De-Brujin algorithm using macaque as an outgroup.

#### Detection of multi-species conserved sequences

Multi-species conserved sequences (MCSs) were detected across the 40-kb *Ush1g*-*Otop2*-*Otop3 *locus using ExactPlus ([35]; http://research.nhgri.nih.gov/projects/exactplus/). Briefly, a line-by-line MultiPipMaker alignment was generated with the high-quality genomic sequence from galago, dog, armadillo, cow, and hedgehog, along with the mouse (build 37; Chr11:115168000-115209000) and human (hg17; Chr17:70408200-70458400) orthologous sequences from the UCSC Genome Browser. Conservation parameters (designated as 7-5-4) were set such that ExactPlus would scan the MultiPipMaker alignment (acgt) file and find blocks of bases (or 'seeds') where a minimum of five species had identical sequences for seven bases; a subsequent base-by-base extension step in either direction was allowed on condition that each extended base was identical in a minimum of four species. Detected MCSs that coincided with UCSC Genome Browser gene annotations, gene predictions, mouse mRNAs, or spliced mouse ESTs were removed to enrich for non-coding MCSs. Subsequently, we intersected the ExactPlus-detected MCSs with MCSs represented on the "PhastCons Conserved Elements, 30-way Vertebrate Multiz Alignment" track in the UCSC Genome Browser (phastConsElement30way; http://genome.ucsc.edu). This track displays conserved sequences detected in alignments of 30 vertebrate sequences using phastCons from the PHAST package [36]; note that the phastConsElement30way default parameters are tuned to detect the top 5% most-conserved sequences in the genome. The intersection of the two data sets revealed 24 shared non-coding MCSs, accounting for ~0.8% of the analyzed sequence. These 24 are considered the most conserved non-coding MCSs across this ~40-kb genomic region. Comprehensive listings of the ExactPlus-identified MCSs and their intersection with phastConsElement30way are available in additional file 13.

#### Phylogenic tree generation

A comprehensive phylogeny of the ODP family in vertebrates and invertebrates has been described elsewhere [12]. Here, we specifically examined the phylogenetic relationships between the multiple amphibian *Otop *genes with respect to the set of three subfamilies (*Otop1*, *-2*, and *-3*) typically found in other vertebrates. In *X. tropicalis*, the *Otop *family consists of eight genes, as determined by a combination of targeted BAC sequencing and database searches. Specifically, six genes were identified and annotated in BAC AC166187 (which was specifically isolated and sequenced for this study), and two genes were identified and annotated in *X. tropicalis *scaffold_441 (genome.ucsc.edu; see additional file 1 for *Otop1a *and *Otop1b *coordinates in the *X. tropicalis *assembly). Thirteen identified *X. tropicalis *ESTs and a full-length cDNA were used to verify the *Otop *gene annotations ([GenBank: CX926385], *Otop1a*; [GenBank: DN050494], non-spliced *Otop2a*; [GenBank: DN023283], *Otop2b*; [GenBank: EL816442], *Otop2c*; [GenBank: DN086801, BX774194, EL846279, DN086800 and CR448138], *Otop3b; *[GenBank: EL846278, BX774945, AL858287 and AL886472], *Otop3c; *and [GenBank: BC155406], full-length *Otop3b*). For *X. laevis*, only a partial gene complement could be identified by literature and database searches, including one EST ([GenBank: EB479546], *Otop3a*) and a full-length cDNA, *Nlo *[20]; also three partial *Nlo *ESTs were identified [GenBank:EB479546, BJ065669 and BJ077411]. A multi-species protein sequence alignment was created that included all known amphibian, mouse, human, and dog Otop sequences. Otop proteins contain 12 trans-membrane domains [12]; for these analyses, the entire intracellular domain and four loops (L5, L6, L8, and L10) were removed from the multi-sequence alignment because of their highly variable length across phyla. The resulting alignment was analyzed using RaxML with a JTT mixed model of amino-acid substitution to generate the Maximum Likelihood (ML) tree. Of the ten inferences generated from ten distinct randomized maximum parsimony (MP) starting trees, four of the resulting phylogenetic trees produced a maximum likelihood value of -8429.55674. The resulting tree was drawn with FigTree (http://tree.bio.ed.ac.uk/software/figtree/).

## List of Abbreviations

(USH): Usher syndrome; (*USH1G*; also *SANS*): USH subtype 1G gene; (SAM): Sterile alpha motif domain; (Otop): otopetrin; (ODP): Otopetrin Domain Protein; (*Nlo*): neural crest-lateral otic vesicle localization gene; (*Zfp509*): zinc finger protein 509; (*Lyar*): Ly1 antibody reactive clone; (*Tmem128*): transmembrane protein 128; (*Drd5*): dopamine receptor 5; (*Slc2a9*): facilitated glucose transporter member 9; (OR): olfactory receptor; (SD): segmental duplication; (TBSD): tumor break-prone segmental duplication; (*USP17*): deubiquitinating enzyme gene; (*Fads6*): fatty acid desaturase domain family member 6; (*C17orf2*): hypothetical protein; (*Cdr21*): cerebellar degeneration-related protein 2-like; (BAC): Bacterial Artificial-Chromosome clone; (FISH): Fluorescence in situ hybridization; (CTCF): CCCTC-binding factor, zinc finger protein; (ChIP-seq): chromatin immunoprecipitation-sequencing; (ES): embryonic stem cells; (MCSs): Multi-species Conserved Sequences; (HRE): human renal epithelial cells; (BJ): skin fibroblasts; (HUVEC): human umbilical vein endothelial cells; (SAEC): small airway epithelial cells; (NHEK): normal human epidermal keratinocytes; (EST): expressed sequence tag; (Oc-22): otoconin-22; (*MYO7A*): myosin VIIa; (*CDH23*): cadherin 23; (*PCDH15*): protocadherin 15; (*js*): *Jackson shaker*; (*tlt*): *tilted*; (*mlh*): *mergulhador*; (*ied*): *inner ear defect*; *(bsk*): *backstroke; *(WGAC): genome assembly comparison; (WSSD): genome shotgun sequence detection; (ML): Maximum Likelihood; (MP): maximum parsimony; (array CGH): Array Comparative Genome Hybridization.

## Authors' contributions

BH and EDG designed the overall study. BH, TMB, FA, and JFR performed all analyses and drafted the manuscript. NISC Comparative Sequencing Program provided sequence data. IH, EEE, DMO, and EDG edited the manuscript. All authors read and approved the final manuscript.

## Supplementary Material

Additional file 1 **UCSC custom track bed file: *Xenopus tropicalis Otop1b*; *Xenopus tropicalis Otop1a***. Click here for file

Additional file 2 ***Table S1***. Pair-wise alignment between the proximal and distal segmental duplications flanking human cytogenetic band 4p16.1-p16.3 Click here for file

Additional file 3 ***Figure S1***. Comparative genomic architecture of the chromosome 4p16.3 region Click here for file

Additional file 4 *Segmental duplication content of the Otop1-proximal and distal regions in the h17 and h19 human genome sequence assemblies; orthologous conservation of the TBSD family in the primate genomes* Click here for file

Additional file 5 ***Figure S2***. Segmental duplication content of the *Otop1*-proximal and *Otop1*-distal regions in the hg17 and hg19 genome assemblies Click here for file

Additional file 6 ***Figure S3***. Distribution of the TBSD segmental duplication family across the human genome Click here for file

Additional file 7 UCSC custom track bed file: *Gasterosteus aculeatus Sansa*; *Gasterosteus aculeatus Sansb*; *Gasterosteus aculeatus Sansc* Click here for file

Additional file 8 UCSC custom track bed file: *Tetraodon nigroviridis Sansa; Tetraodon nigroviridis Sansb; Tetraodon nigroviridis Sansc* Click here for file

Additional file 9 UCSC custom track bed file: *Oryzias latipes Sansa; Oryzias latipes Sansab; Oryzias latipes Sansac;* Click here for file

Additional file 10 UCSC custom track bed file: *Takifugu rubripes Sansa*; *Takifugu rubripes Sansb: Takifugu rubripes Sansc* Click here for file

Additional file 11 UCSC custom track bed file: *Danio rerio Sansb; Danio rerio Sansc* Click here for file

Additional file 12 ***Figure S4***. Comparative analysis of the Ush1g proteins in five fish species Click here for file

Additional file 13 UCSC custom track bed file: ExactPlus 7-5-4 MCSs; ExactPlus 7-5-4 overlaps phastConsElement30way MCSs Click here for file
