# Supplementary material for: Lineage-specific evolution of the vertebrate Otopetrin gene family revealed by comparative genomic analyses
Source: BMC Evol Biol. 2011 Jan 24;11:23. doi: 10.1186/1471-2148-11-23 (PMC3038909; doi:10.1186/1471-2148-11-23)
Supplement: Additional file 3 — Figure S1. Comparative genomic architecture of the chromosome 4p16.3 region [file 1471-2148-11-23-S3.PDF]

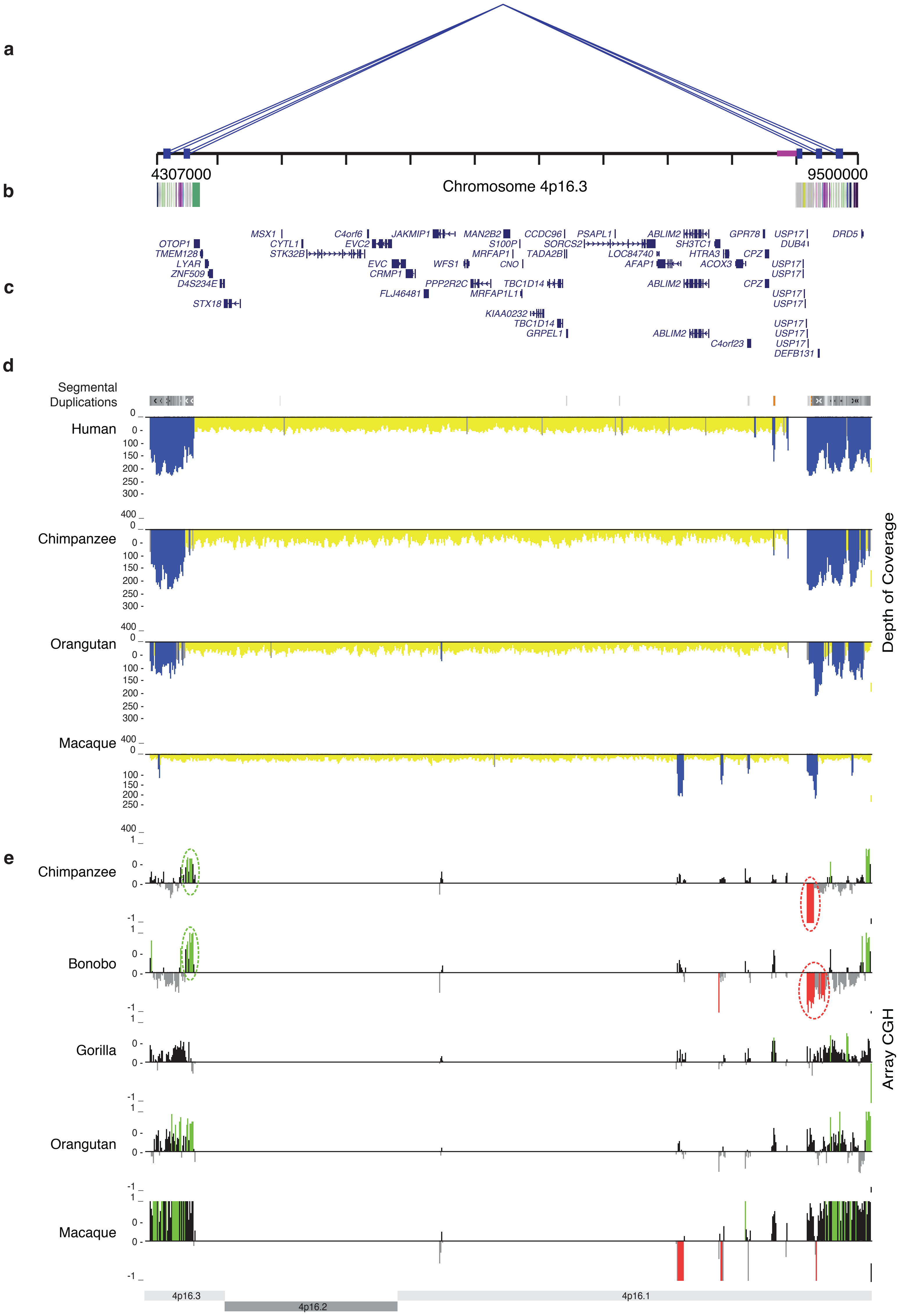


**Figure S1.** *Comparative genomic architecture of the chromosome 4p16.3 region among primates.*

**(a)** The paralogy of a number of large (≥10 kb), highly similar (≥95% identical) duplicated regions flanking the inverted 5-Mb region is highlighted with joining blue lines. **(b)** Underlying duplicon structures, where different color lines represent cytogenetic band locations of ancestral duplicons. **(c)** RefSeq gene track from UCSC Genome Browser. **(d)** Whole-genome shotgun (WGS) depth-of-coverage analysis using sequence reads generated from whole-genome sequencing of the human, chimpanzee, orangutan, and macaque genomes. Regions of excess read coverage (≥ mean +3 standard deviation) are indicative of duplicated segments (colored in blue). The vertical axis refers to the number of WGS reads mapped in a certain position of the human genome. **(e)** Array CGH results for chimpanzee, bonobo, gorilla, orangutan, and macaque genomic regions using human as the reference. Probes with color bars (red or green) correspond to signal intensity beyond 1.5 standard deviation of the normalized average [30]. The dashed green circles highlight excess copy number of a ~60-kb duplicated segment containing *OTOP1*-like and *TMEM128*-like sequences in human *versus* chimpanzee and bonobo. The dashed red circles highlight excess copy number of the RS447 megasatellite in bonobo and chimpanzee *versus* human.
