# Supplementary material for: Lineage-specific evolution of the vertebrate Otopetrin gene family revealed by comparative genomic analyses
Source: BMC Evol Biol. 2011 Jan 24;11:23. doi: 10.1186/1471-2148-11-23 (PMC3038909; doi:10.1186/1471-2148-11-23)
Supplement: Additional file 4 — Segmental duplication content of the Otop1-proximal and distal regions in the h17 and h19 human genome sequence assemblies; orthologous conservation of the TBSD family in the primate genomes [file 1471-2148-11-23-S4.DOC]

*Segmental duplication content of the Otop1-proximal and distal regions in the h17 and h19 human genome assemblies*

The TBSD (tumor break-prone) family of segmental duplication (SD) is specific to the great apes, emerging and expanding in hominid genomes since the divergence of the human, chimpanzee, and orangutan common ancestor from the macaque lineage roughly 12-16 million years ago. The number of SD belonging to this family annotated in the human genome has increased with each new released sequence assembly; specifically, there are 14, 17, and 18 SD copies annotated in the hg16, hg17, and hg19 releases, respectively ([1] and present study). For instance, the *OTOP1*-distal flanking region contains one SD in hg17 but two tandemly arranged SD in hg19 (see additional file 5); this is not unexpected since the rare gaps remaining in the assembled human genome sequence are enriched for structurally complex sequences [2]. Note that the 17 copies of the SD present in hg17 are clustered on chromosomes 3, 4, 7, 8, 11, 12, and 16, and mostly reside within subtelomeric and pericentromeric regions (see additional file 6 for additional details).

*Orthologous conservation of the TBSD family in the primate genomes*

Comparisons between the human and chimpanzee genome sequences revealed that orthologous SD resided at the expected locations in the chimpanzee genome, with the exception of a SD on human chromosome 11q23 (which is unique to humans [1]). Also, the chimpanzee genomic regions orthologous to the subtelomeric portions of human chromosomes 8p23.1 and 4p16.1 could not be fully evaluated, as these portions of the assembled chimpanzee genome sequence contain numerous gaps. Transcribed sequences within the TBSD family SD include an anonymous cDNA (*BC04282*) and a new member of the *FAM86* family. *FAM86* genes encode proteins containing the S-adenosyl-L-methionine-dependent methyltransferase superfamily domain, which may play a role in gene regulation and differentiation [3]. Consistent with the known evolutionary history of the TBSD family, we failed to detect most orthologous sequences in the orangutan or rhesus macaque genomes. For example, we found 13 *FAM86* genes, one functional copy of *UNC93B1*, and six *ΨUNC93B1* pseudogenes in the human and chimpanzee genomes, but only one *FAM86* gene and one *UNC93B1* gene in the macaque genome (located on chromosomes 20 and 14 in regions that are orthologous human chromosomes 16p13.3 and 11q13.2, respectively).

**References**

1. Mehan MR, Almonte M, Slaten E, Freimer NB, Rao PN, Ophoff RA: **Analysis of segmental duplications reveals a distinct pattern of continuation-of-synteny between human and mouse genomes**. *Hum Genet* 2007, **121**(1):93-100.

2. Platzer M: **The human genome and its upcoming dynamics**. *Genome Dyn* 2006, **2**:1-16.

3. Darai-Ramqvist E, Sandlund A, Muller S, Klein G, Imreh S, Kost-Alimova M: **Segmental duplications and evolutionary plasticity at tumor chromosome break-prone regions**. *Genome Res* 2008, **18**(3):370-379.
