# Supplementary material for: Lineage-specific evolution of the vertebrate Otopetrin gene family revealed by comparative genomic analyses
Source: BMC Evol Biol. 2011 Jan 24;11:23. doi: 10.1186/1471-2148-11-23 (PMC3038909; doi:10.1186/1471-2148-11-23)
Supplement: Additional file 5 — Figure S2. Segmental duplication content of the Otop1-proximal and Otop1-distal regions in the hg17 and hg19 genome assemblies [file 1471-2148-11-23-S5.PDF]

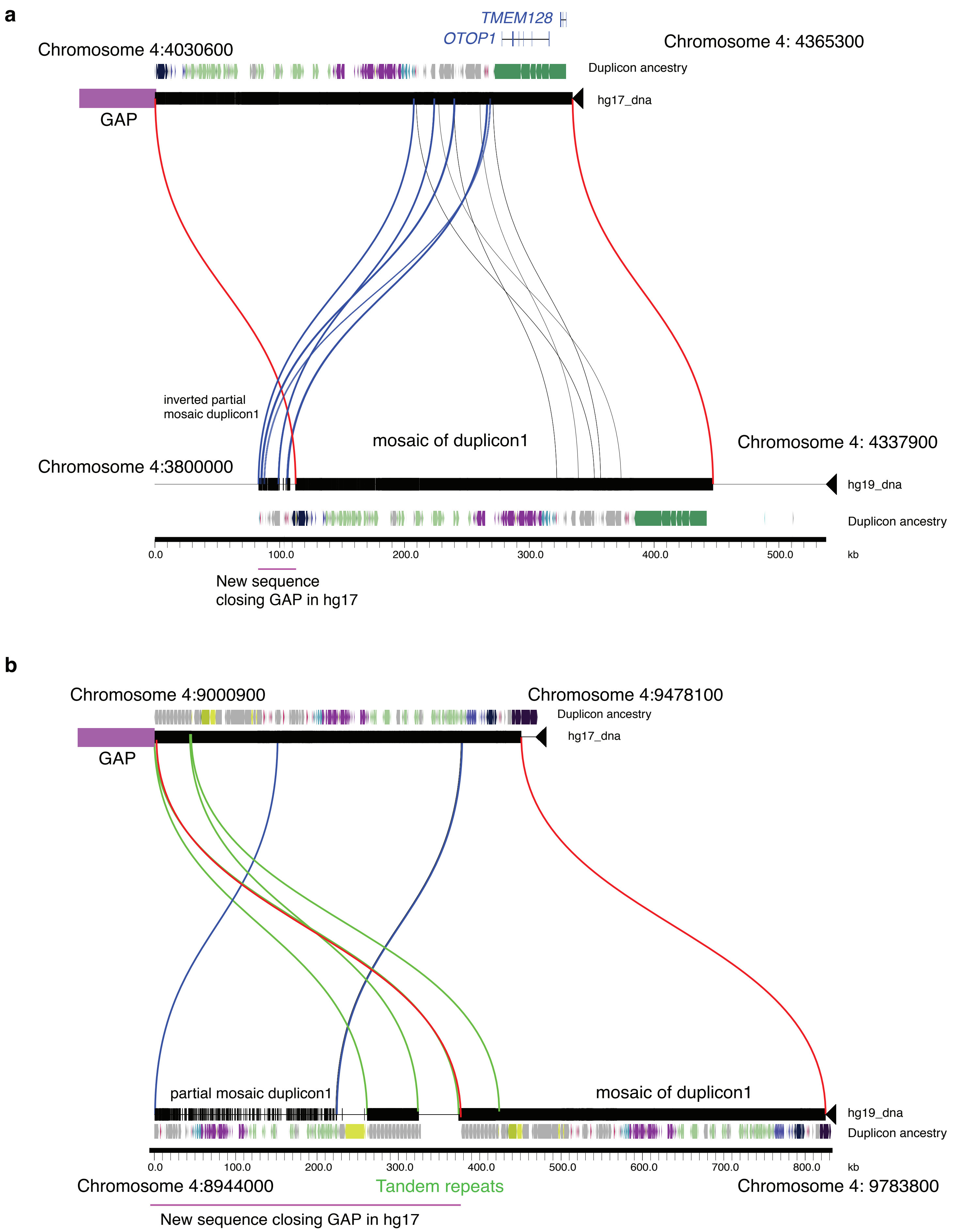

Figure S2. *Segmental duplication content of the Otop1-proximal and -distal regions in the hg17 and hg19 genome assemblies.*

The *Otop1*-proximal **(a)** and -distal **(b)** duplicon genomic regions were extracted from the hg17 and hg19 human assemblies and aligned with Miropeats [63], which identifies regions of sequence similarity between the assemblies and displays. This similarity information is depicted graphically in the positional context of the sequence as black solid bars delineated by joining lines, which are color-coded for clarity as follows: mosaic duplicon 1 present in both assemblies, red joining lines; mosaic duplicon 1 annotated in the new hg19 sequence closing a previous sequencing gap in hg17, blue joining lines; and additional tandem repeat duplications in between large mosaic duplicons, green joining lines [(**b**) panel only]. Pertinent gene annotations and ancestral duplicon composition of each duplicon (obtained from DupMasker annotations [28]) are also shown. Note that the *OTOP1*-distal flanking region in **(b)** contains one duplicon in hg17 but two tandemly arranged duplicons in hg19.
