# Supplementary material for: Lineage-specific evolution of the vertebrate Otopetrin gene family revealed by comparative genomic analyses
Source: BMC Evol Biol. 2011 Jan 24;11:23. doi: 10.1186/1471-2148-11-23 (PMC3038909; doi:10.1186/1471-2148-11-23)
Supplement: Additional file 6 — Figure S3. Distribution of the TBSD segmental duplication family across the human genome [file 1471-2148-11-23-S6.PDF]

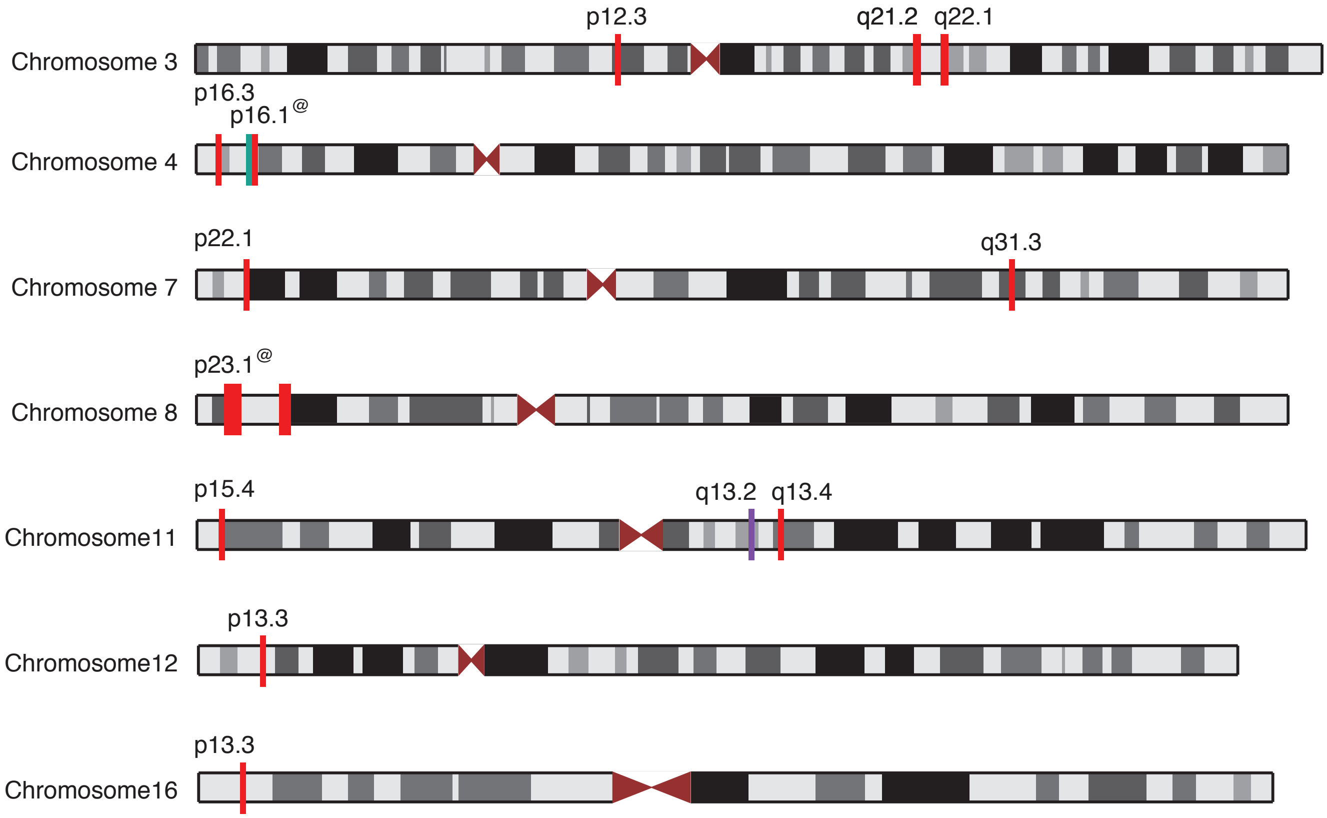

Figure S3. *Distribution of the TBSD family across the human genome.*

The ideograms of human chromosomes 3, 4, 7, 8, 11, 12, and 16 are shown, not drawn to scale. Relevant cytogenetic bands are labeled. The TBSD [26] family of 18 large (~260-kb) segmental duplications that are highly homologous (>95% sequence identity) are depicted as short vertical bars color coded as follows: red, segmental duplications found in the hg17 human genome sequence assembly with confirmed orthologs in the chimpanzee genome assembly (build2); and green and purple lines represent a new duplicon annotated based on the hg19 assembly and a duplicon unique to *Homo sapiens* [30], respectively. Note the concentration of segmental duplications near subtelomeric and pericentromeric regions. Inversion polymorphisms on human chromosomes 4p16 and 8q23 thought to trigger the t(4;8)(p16;p23) translocation in double heterozygous are indicated with an @ ([15, 44, 45]).
