# Supplementary material for: Lineage-specific evolution of the vertebrate Otopetrin gene family revealed by comparative genomic analyses
Source: BMC Evol Biol. 2011 Jan 24;11:23. doi: 10.1186/1471-2148-11-23 (PMC3038909; doi:10.1186/1471-2148-11-23)
Supplement: Additional file 12 — Figure S4. Comparative analysis of the Ush1g proteins in five fish species [file 1471-2148-11-23-S12.PDF]

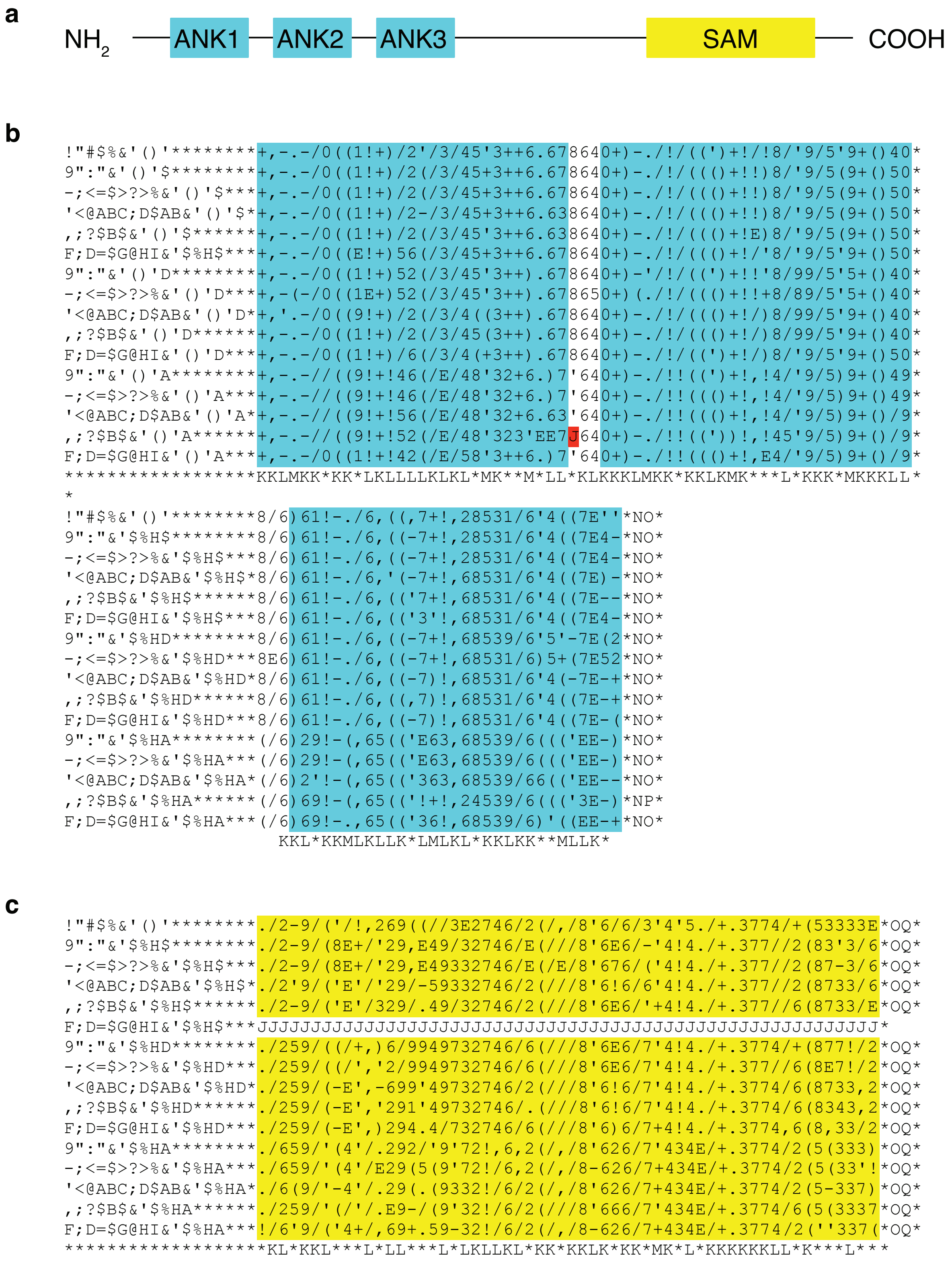

Supplementary Figure 4. *Comparative analysis of the Ush1g proteins in five fish species.*

(a) Schematic representation of the human USH1G protein. The known functional domains include three ankyrin repeats (ANK1-ANK3) and sterile alpha motif (SAM), represented as turquoise and yellow boxes, respectively. **(b)** ANK and **(c)** SAM multi-species sequence alignments for human USH1G and its three orthologs (Ush1ga, Ush1gb, and Ush1gc) in five fish species. For any given amino acid residue in the multi-sequence alignments, identity with the human residue is marked with an asterisk (*); (:) indicates conservative amino acid change. The sequence encoding the SAM domain in zebrafish Ush1ga lies within a sequence gap the zebrafish genome assembly and is represented by dashes. The dash highlighted in red for medaka Ush1gc ANK1 indicates a putative frameshift, resulting in a premature stop codon. See additional files 7, 8, 9, 10, and 11 for the original data used to perform this analysis.
